# Metabolic Profile of Senegalese Sole (*Solea senegalensis*) Muscle: Effect of Fish–Macroalgae IMTA-RAS Aquaculture

**DOI:** 10.3390/molecules30122518

**Published:** 2025-06-09

**Authors:** Flaminia Cesara Marincola, Chiara Palmas, Miguel A. Lastres Couto, Isabel Paz, Javier Cremades, José Pintado, Leonardo Bruni, Gianfranco Picone

**Affiliations:** 1 Department of Chemical and Geological Sciences, University of Cagliari, Monserrato, 09042 Cagliari, Italy; flaminia@unica.it (F.C.M.); c.palmas10@studenti.unica.it (C.P.); 2 Instituto Galego de Formación en Acuicultura (IGAFA), Xunta de Galicia, 36626 Illa de Arousa, Spain; miguel.anxo.lastres.couto@xunta.gal (M.A.L.C.); isabel.paz.boubeta@xunta.gal (I.P.); 3 Biología Costera (BioCost), Centro de Investigaciones Científicas Avanzadas (CICA), Universidad de A Coruña, 15001 A Coruña, Spain; javier.cremades@udc.es; 4 Instituto de Investigacións Mariñas—Institute of Marine Research (IIM-CSIC), Eduardo Cabello 6, 36208 Vigo, Spain; pintado@iim.csic.es (J.P.); leonardobrunifi@gmail.com (L.B.); 5 Department of Agricultural and Food Sciences (DISTAL), University of Bologna, Viale G. Fanin 40, 40127 Bologna, Italy

**Keywords:** recirculating aquaculture system, integrated multi-trophic aquaculture, sustainability, NMR-based metabolomics, probiotics, *Ulva ohnoi*

## Abstract

The aquaculture sector is essential for meeting seafood demand while ensuring sustainability. It involves farming fish, mollusks, crustaceans, other invertebrates, and algae in controlled environments, helping to conserve marine resources and reduce ecological pressures. Sustainable practices, such as an integrated multitrophic recirculating aquaculture system (IMTA-RAS) with fish and seaweed, can minimize the environmental impact of fish aquaculture. However, the impact of the introduction of macroalgae on the fish muscle metabolism has not been studied. This research examines the impact of growing Senegalese sole (*Solea senegalensis*) together with sea lettuce (*Ulva ohnoi*) on fish metabolism using high-resolution ^1^H-NMR-based metabolomics. Three farming systems were compared. These were E_1_, a recirculating aquaculture system (RAS); E_2_, an IMTA-RAS integrating *U. ohnoi* for biofiltration; and E_3_, an IMTA-RAS with *U. ohnoi* and *Phaeobacter* sp. strain 4UAC3, a probiotic bacterium isolated from wild *U. australis* known to counteract fish pathogens. A metabolomic analysis revealed that energy metabolism was enhanced in IMTA-RAS and even more in IMTA-RAS-*Phaeobacter*–grown fish, increasing overall metabolic activity. These results indicate that the presence of the algae with the probiotic had a clear impact on the physiological state of the fish, and this deserves further investigation. This study contributes to the understanding of the physiological responses of fish to innovative aquaculture practices, supporting the development of more sustainable and efficient management that reduces the environmental impact and increases fish health and welfare.

## 1. Introduction

Goal 14 of the United Nations 2030 Agenda focuses on “Life Below Water”, aiming to promote the conservation and responsible management of oceans, seas, and marine resources for sustainable development. Specifically, Goal 14 emphasizes the protection of marine ecosystems, a reduction in marine pollution, the sustainable management of marine resources, and the preservation of marine biodiversity [1,2,3]. According to the FAO [4], when developed sustainably, aquaculture can play a pivotal role in advancing this goal by enhancing food production, ensuring food security and nutrition, and providing significant socio-economic benefits while promoting environmentally responsible practices [5].

To align with these sustainability goals, recent advances in aquaculture have led to sustainable systems like recirculating aquaculture systems (RASs) and integrated multitrophic aquaculture (IMTA). RASs minimize resource use and waste production by continuously filtering and reusing water [6], while IMTA enhances efficiency by integrating species at different trophic levels, using fish waste to nourish organisms like algae and mollusks [7]. Combining RAS and IMTA creates a balanced, sustainable cycle that improves water quality, promotes ecological stability, reduces the environmental impact, and supports a more balanced and productive aquaculture system [8,9].

*Ulva ohnoi*, a green macroalga belonging to the *Ulvaceae* family [10], is endemic to Japan and predominantly distributed in tropical and subtropical regions. It is characterized by a flat, thin, and semi-transparent thallus, morphologically resembling a lettuce leaf, which justifies its common designation as “sea lettuce”. Due to its high nutrient uptake efficiency, particularly in nitrogen-enriched environments, as well as its ability to thrive at temperatures compatible with Senegalese sole (*Solea senegalensis*) aquaculture, *U. ohnoi* has been identified as a suitable candidate for nutrient biofiltration in IMTA-RASs for sole cultivation [11,12]. Furthermore, *U. ohnoi* experimentally colonized with *Phaeobacter* sp. 4UAC3, a bacterium isolated from *U. australis* with antagonistic activity against pathogenic *Vibrio* species due to the production of tropodithietic acid (TDA) [13], has recently demonstrated potential as an alternative to traditional disease control methods in IMTA-RASs, offering the added advantage of reducing fish mortality in aquaculture settings [14,15,16]. However, the impact of the introduction of macroalgae or a probiotic on fish metabolism has not been studied.

Metabolomics is a powerful high-throughput technology that enables the comprehensive analysis of metabolites within an organism, offering valuable insights into its physiological state. Initially developed for biomedical research [17,18,19,20,21], metabolomics has rapidly expanded into a wide range of disciplines, including environmental science, agriculture, and aquaculture [22]. In aquaculture, metabolomic applications have demonstrated vast potential for addressing challenges across the entire production chain. Its applications range from monitoring the health and welfare of farmed species [23] to evaluating nutritional status [24] and optimizing feed formulations to improve growth performance and feed efficiency [25,26]. Moreover, metabolomic tools have proved to be useful in quality control by identifying markers associated with freshness, spoilage, or product differentiation [27]. An additional and increasingly relevant area of application lies in evaluating the effectiveness of probiotics and functional feed additives, particularly in modulating immune responses and enhancing disease resistance [28,29]. These strategies represent sustainable alternatives to the use of antibiotics in aquaculture, and metabolomics can provide a molecular-level understanding of their impact.

The aim of this study was to investigate the metabolomics profile of the muscles of Senegalese sole reared under three different aquaculture conditions. These were: E_1_, a RAS; E_2_, an IMTA-RAS with *U. ohnoi* (IMTA-RAS); and E_3_, an IMTA-RAS with *U. ohnoi* inoculated with the probiotic *Phaeobacter* sp. 4UAC3 (IMTA-RAS-Phaeobacter). Muscle tissue, a metabolically active and nutritionally relevant compartment, was chosen for the analysis as it reflects both systemic physiological responses and the nutritional quality of the final product. The use of ^1^H-NMR spectroscopy enabled the untargeted, high-resolution detection of a broad range of metabolites, offering a holistic view of the biochemical alterations induced by the different rearing strategies. This approach provided key insights into how the integration of macroalgae and probiotics into RASs could modulate fish metabolism. The potential implications for growth performance, health status, and product quality will be extensively explored in other papers from the same project, which assesses aspects such as fish welfare and quality, *Ulva* performance, the system microbiome, and water parameters.

## 2. Results

Fish biometrics after six weeks of growth (T_f_) did not significantly differ between the three experimental conditions (Table 1), although a trend of increased performance from E_1_ to E_3_ was apparent. One fish in E_1_ and two in E_3_ died between T_i_ (after the acclimatization period) and T_f_; the remaining fish did not show signs of discomfort.

**Figure 1:**
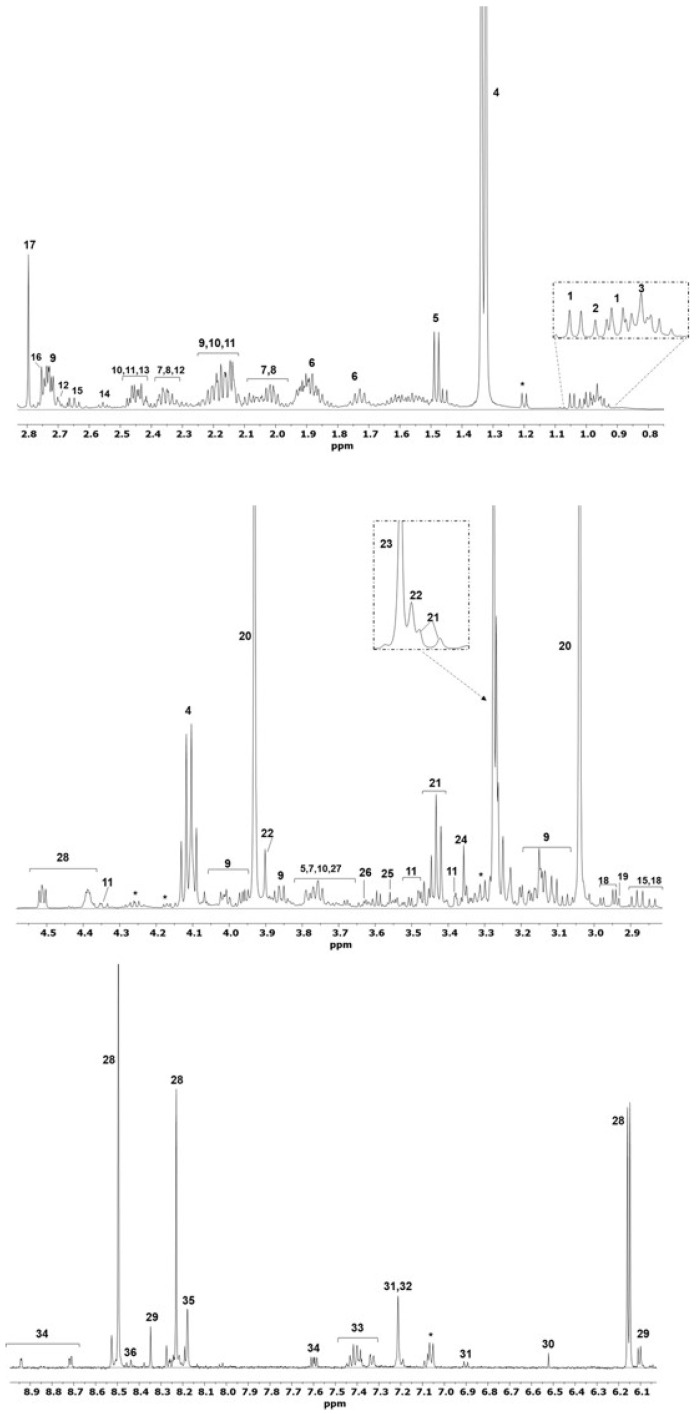
Expanded regions of a representative ^1^H-NMR spectrum of the aqueous extract from *S. senegalensis* muscle. Key: 1. valine, 2. leucine, 3. isoleucine, 4. lactate, 5. alanine, 6. lysine, 7. glutamate, 8. proline, 9. cystathionine, 10. glutamine, 11. trans-4-hydroxy-L-proline, 12. malate, 13. succinate, 14. β-alanine, 15. aspartate, 16. dimethylamine, 17. methylguanidine, 18. asparagine, 19. dimethylglycine, 20. creatine, 21. taurine, 22. betaine, 23. trimethylamine N-oxide, 24. methanol, 25. glycine, 26. glycerol, 27. O-acetylcarnitine, 28. nucleotides, 29. inosine, 30. fumarate, 31. tyrosine, 32. histidine, 33. phenylalanine, 34. niacinamide, 35. adenine, and 36. formate. Unassigned signals are marked with an asterisk.

Figure 1 shows a representative spectrum of the aqueous extract from the muscle of *S. senegalensis*. Overall, the signals of 36 metabolites were identified (Figure S1). The region between 0.5 and 4.5 ppm encompassed the signals of amino acids (alanine, leucine, isoleucine, valine, β-alanine, glycine, asparagine, glutamate, glutamine, and proline), succinate, trimethylamine N-oxide (TMAO), lactate, malate, cystathionine, creatinine, creatine, phosphocreatine, betaine, O-acetylcarnitine, dimethylamine (DMA), *N*,*N*-dimethylglycine (DMG), and trans-4-hydroxy-L-proline. The region between 6.0 and 8.8 ppm included the signals of aromatic amino acids (phenylalanine, histidine, and tyrosine), formate, fumarate, key nitrogenous bases such as adenosine monophosphate (AMP) and inosine monophosphate (IMP), and nicotinamide.

**Figure 2:**
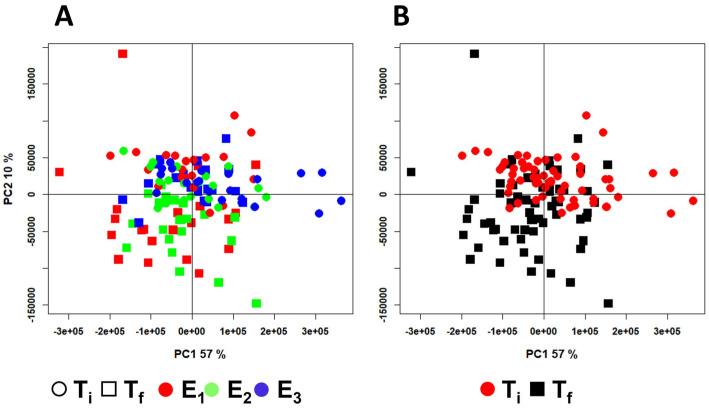
PCA score plots of the model built using the mean-centered, binned ^1^H-NMR spectral dataset of *S. senegalensis* muscle extracts. PC1 and PC2 account for 57% and 10% of the total variance, respectively. The scores are color-coded according to (**A**) the experimental conditions (E_1_: a RAS; E_2_: an IMTA-RAS with *U. ohnoi*; and E_3_: an IMTA-RAS with *U. ohnoi* inoculated with the probiotic *Phaeobacter* sp. 4UAC3), and (**B**) the time points (T_i_, after the acclimatation, and T_f_, six weeks later at the conclusion of each trial).

To examine the potential metabolic responses of Senegalese sole to the three different aquaculture conditions, the ^1^H-NMR spectral dataset was initially analyzed using an exploratory PCA. Figure 2 displays the corresponding score plot (PC1 vs. PC2), accounting for almost 67% of the total variance. Although the three groups partially overlapped, a moderate divergence was noticeable, particularly for E_3_ samples along PC1. This pattern suggested that the combination of macroalgae and probiotic could have elicited a different metabolic profile in the muscle tissue compared with the other treatments. In contrast, the closer proximity of the E_2_ scores to those of E_1_ indicated that *U. ohnoi* alone induced a metabolic response more similar to the RAS-only condition, suggesting only moderate alterations. The separation among the scores based on the sampling time (T_i_ and T_f_) appeared to be more pronounced (Figure 2B), reflecting the cumulative impact of the rearing conditions over the experimental period. A few scores were identified as outliers; among these, only two from the T_i_ group were excluded from further analyses as a visual inspection of the spectrum revealed a significant baseline distortion. In addition, objective criteria such as Hotelling T^2^; and DModX were applied to justify their exclusion from the final principal component analysis (Figure S1).

**Figure 3:**
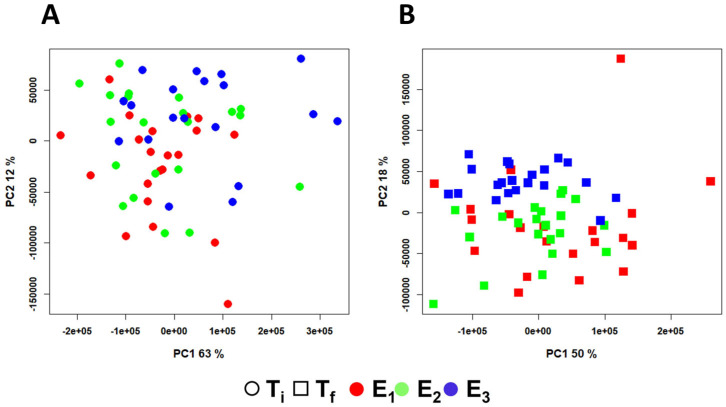
PCA score plots obtained from the mean-centered, binned ^1^H-NMR spectral dataset of *S. senegalensis* muscle extracts of samples collected (**A**) at the initial time point (T_i_) and (**B**) at the final time point (T_f_). In both plots, scores are color-coded according to the experimental conditions: E_1_ (RAS), E_2_ (IMTA-RAS with *U. ohnoi*), and E_3_ (IMTA-RAS with *U. ohnoi* inoculated with the probiotic *Phaeobacter* sp. 4UAC3). PC1 and PC2 account for 63% and 12% of the total variance, respectively, in model (**A**) and 50% and 18% in model (**B**).

To further explore the differences among the three experimental conditions and their effect on the metabolomic profile of sole muscle, two additional PCA models were constructed using only the spectra from samples collected at the same time points. Figure 3 displays the PC1 vs. PC2 score plots for these models, with the scores color-coded according to the experimental conditions. As shown in Figure 3A, the scores from the T_i_ sampling across the three experiments highly overlapped. This indicated that the fish shared a common metabolic baseline, confirming the absence of significant pre-existing differences among the experimental conditions at the start of the trial. Although no clear group separation was evident at the start of the trial, a trend toward divergence became apparent at T_f_ (Figure 3B), particularly for the E_3_ group. This suggested a potential cumulative metabolic effect associated with the combined presence of macroalgae and the probiotic. In contrast, the metabolic profiles of E_1_ and E_2_ remained more similar, implying a more moderate response to algae alone.

**Figure 4:**
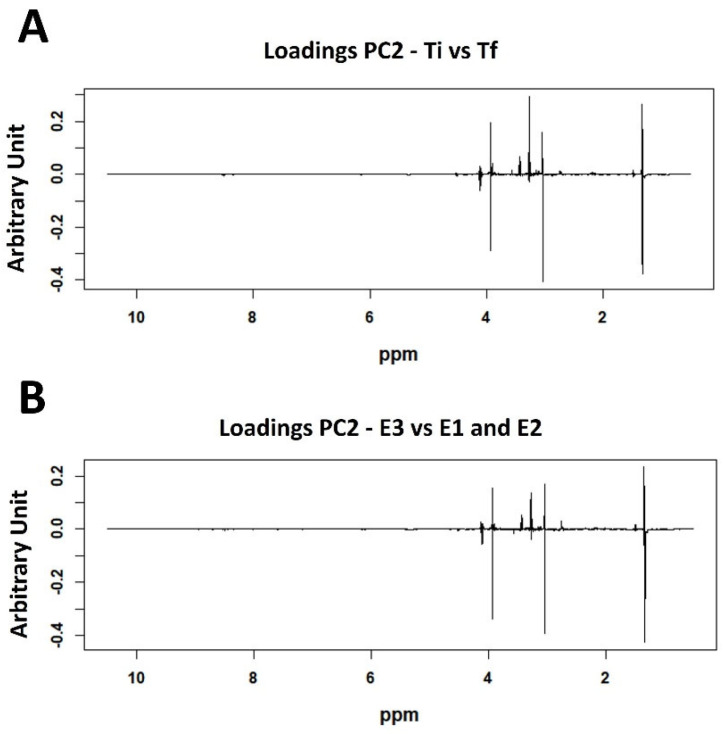
PC2 loading plots obtained from the PCA performed on the normalized and binned ^1^H-NMR spectra. Panel (**A**) corresponds with the model shown in the score plot of Figure 2, comparing samples collected at the initial (T_i_) and final (T_f_) time points. Panel (**B**) refers to the model shown in Figure 3B, comparing only the three experimental conditions at T_f_. Positive loadings indicate spectral regions associated with higher metabolite levels in (**A**) samples collected at Ti across all experimental groups and (**B**) the E_3_ group at T_f_. Conversely, negative loadings indicate metabolites more abundant in (**A**) samples collected at T_f_ and (**B**) the E_2_ group at T_f_.

The analysis of the corresponding loading plots of the PCA represented in Figure 2 allowed for the identification of the metabolites responsible for the separation of the samples according to the time factor (Figure 4A). The results highlight that the NMR spectra of the samples collected at the final time point across all experiments showed higher levels of lactate, TMAO, creatine, and phosphocreatine, along with lower levels of taurine.

As no significant differences were detected between the experimental groups at T_i_, further insights into the effects of the three experimental conditions were obtained by analyzing the data collected at the final time point (Figure 4B). Thus, the relative levels of various metabolites in Senegalese sole muscle were compared using area normalization and evaluated using the Kruskal–Wallis test. Only metabolites with well-resolved and isolated signals in the ^1^H-NMR spectra, allowing reliable integration, were included in the analysis. The results are presented in Table 2, which reports the unadjusted *p*-values, epsilon-squared (ε^2^) effect sizes, and FDR-corrected *p*-values.

Among the 30 metabolites analyzed, 24 exhibited statistically significant differences across the experimental groups. In particular, DMA showed the strongest effect (*q* = 0.002; ε^2^ = 0.813) followed by taurine (*q* = 0.002; ε^2^ = 0.588) and tyrosine (*q* = 0.002; ε^2^ = 0.601), indicating a substantial variation in their levels among the three trials. Several other metabolites such as cystathionine, trans-4-hydroxy-L-proline, inosine, alanine, phenylalanine, and methylguanidine also showed highly significant differences with large effect sizes (ε^2^ > 0.14), suggesting a marked influence of the experimental conditions on these metabolic pathways. Conversely, some metabolites, including AMP/IMP, creatine, lactate, and TMAO, showed no significant variation (*p* > 0.05), indicating the relative stability of these compounds across the different conditions tested.

**Figure 5:**
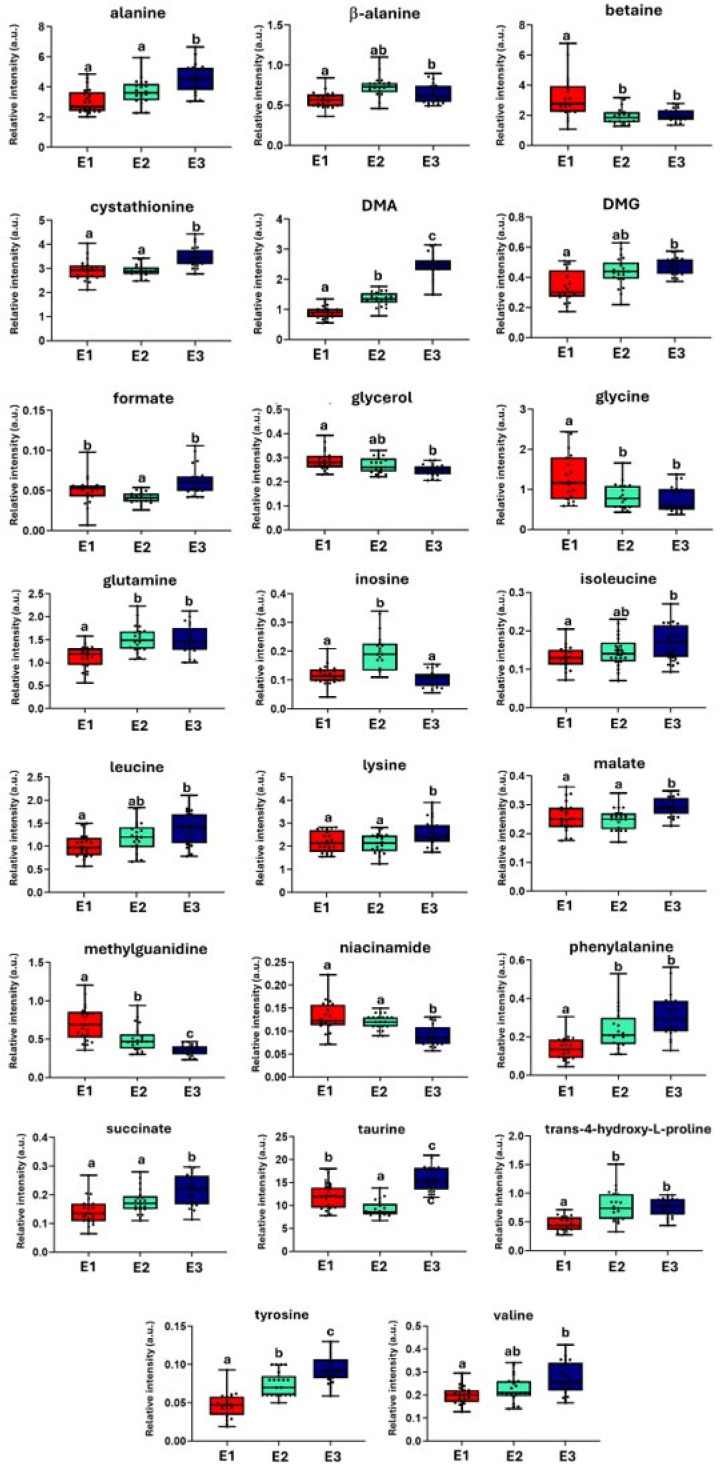
Box plots showing the relative intensity (a.u.) of selected metabolites in muscle tissue of *S. senegalensis* reared under the following three different aquaculture conditions. Colors correspond to the different experimental groups as labeled: red, E_1_ (RAS); cyano, E_2_ (IMTA-RAS with *U. ohnoi*); blue, E_3_ (IMTA-RAS with *U. ohnoi* inoculated with the probiotic *Phaeobacter* sp. 4UAC3). Data refer to samples collected at the final time point. Each box represents the interquartile range (IQR; 25th–75th percentile), with the horizontal line indicating the median. Whiskers extend to the smallest and largest values within 1.5 × IQR. Different lowercase letters above the boxes indicate statistically significant differences between groups (*p* < 0.05; Kruskal–Wallis test followed by pairwise comparisons using the Dwass–Steel–Critchlow–Fligner test). Shared letters indicate no significant difference.

Figure 5 presents the relative intensities of the 24 metabolites in the muscle of *S. senegalensis* across the three experimental conditions. Several metabolites exhibited a progressive increase or decrease from E_1_ to E_3_. In particular, metabolites such as DMA, taurine, and tyrosine showed significantly higher levels in E_3_ compared with E_1_, with E_2_ displaying intermediate values. These trends suggested a gradual metabolic shift, likely enhanced by the addition of both *U. ohnoi* and the probiotic *Phaeobacter* sp. 4UAC3 in the E_3_ condition. Conversely, compounds such as betaine, niacinamide, glycerol, glycine, and methylguanidine progressively decreased from E_1_ to E_3_. For some metabolites, E_3_ was significantly different from both E_1_ and E_2_, while the latter two remained similar (e.g., lysine, malate, and succinate).

## 3. Discussion

The present study is part of a comprehensive project assessing the effects on fish welfare, growth, and quality; *Ulva* growth and quality; the system microbiome; and the water parameters of three aquaculture systems with increasing levels of complexity; namely, a conventional RAS (E_1_), an IMTA-RAS integrating the green macroalga *U. ohnoi* (E_2_), and an IMTA-RAS supplemented with *U. ohnoi* inoculated with the probiotic *Phaeobacter* sp. 4UAC3 (E_3_).

We focused on the metabolic response of *S. senegalensis* muscle to these treatments. The results indicated treatment-related differences in muscle metabolic profiles, as revealed by multivariate (PCA) and univariate (Kruskal–Wallis) analyses.

The concentration of several organic acids significantly varied across the experimental groups, indicating substantial changes in the energy metabolism associated with different rearing strategies. Succinate levels notably increased from E_1_ to E_3_, suggesting an accumulation of this TCA cycle intermediate in fish reared in the *U. ohnoi* and *Phaeobacter* sp.-enriched system. Similarly, the malate concentration rose significantly in E_3_. Lactate levels, on the other hand, remained relatively stable, suggesting that anaerobic glycolysis was not differently activated under any condition.

In aquaculture contexts, succinate accumulation is frequently associated with environmental stressors, including hypoxia or imbalanced nutrition [30,31]. However, in this study, the concurrent rise of malate alongside stable lactate pointed toward a functional metabolic remodeling rather than a pathological response. As highlighted by Liu et al. [32], fish exposed to a RAS can undergo adaptive metabolic adjustments that help to maintain muscle integrity under sustained environmental stimuli. Such metabolic reorganization likely reflected increased biosynthetic or energetic demands under E_3_ conditions. This was supported by the discovery of upregulated branched-chain and aromatic amino acids, such as phenylalanine and tyrosine, compounds involved in muscle remodeling and immunity [33]. Moreover, elevated malate levels could be linked to an upregulation of anaplerotic reactions or compensatory mitochondrial activity, a phenomenon also described in exercise-trained fish [34].

The amino acid profile revealed several treatment-dependent trends. Alanine, glutamine, phenylalanine, and tyrosine significantly increased from E_1_ to E_3_, suggesting intensified protein turnover and nitrogen metabolism. This trend aligned with previous reports demonstrating that amino acid accumulation supports structural and immune responses under intensified rearing conditions [33]. Additionally, trans-4-hydroxy-L-proline, a known marker of collagen turnover and connective tissue remodeling, increased in E_2_ and E_3_ compared with E_1_. This suggests that the co-cultivation of *Ulva*, even without the probiotic, could have promoted structural remodeling processes in the muscle tissue, potentially reflecting enhanced tissue maintenance or growth under improved environmental conditions. Branched-chain amino acids (leucine, isoleucine, and valine) also increased across the groups, reinforcing the notion of muscle adaptation [35]. Interestingly, the observed decrease in glycine levels from E_1_ to E_3_ seemed to contrast with the patterns seen for other metabolites biochemically linked to glycine metabolism. Glycine can be synthesized from multiple precursors, including serine, 4-hydroxyproline, choline, and betaine, via distinct metabolic pathways [36]. In fish, as in terrestrial animals, these biosynthetic routes ensure an adequate glycine supply to support essential functions such as protein synthesis (especially collagen), glutathione production, heme biosynthesis, purine formation, and the generation of glycine-conjugated bile salts and creatine [37]. In this context, the concurrent increase in trans-4-hydroxy-L-proline and decrease in glycine could reflect an enhanced conversion of hydroxyproline to glyoxylate and downstream intermediates, with glycine being rapidly utilized for anabolic processes rather than accumulating in a free form. This dynamic was further supported by the changes observed in the levels of betaine, another glycine precursor via methylation pathways, and its metabolite dimethylglycine (DMG): although betaine decreased, DMG increased in E_3_. As betaine is both a methyl donor and an osmoprotectant [38], its decline, accompanied by DMG accumulation, suggested enhanced demethylation activity, indicative of an increased methyl group turnover. This shift possibly reflected greater biosynthetic or regulatory demands under the more complex rearing conditions of the macroalgae–probiotic system. Notably, both betaine and DMG are also linked to glycine metabolism, and their opposing trends implied that methyl donors were being rerouted toward functions other than glycine production, possibly to support increased metabolic plasticity. Therefore, the drop in glycine levels in E_3_ might not indicate a reduction in its synthesis but rather an increased demand and rapid utilization under the more metabolically active conditions promoted by the combined macroalgae–probiotic treatment. This interpretation was consistent with the broader metabolic remodeling observed in E_3_, characterized by elevated amino acid levels and energy-related intermediates.

Additional metabolites such as cystathionine, niacinamide, DMA, and glycerol provided further insights into the fish physiology. Cystathionine levels progressively increased from E_1_ to E_3_, while no significant difference was observed between E_1_ and E_2_. This trend suggested the potential activation of the trans-sulfuration pathway in E_3_, supporting methionine metabolism and glutathione synthesis, key components in maintaining redox homeostasis [39,40]. The lack of change in E_2_ compared with E_1_ implied that *U. ohnoi* alone may not be sufficient to stimulate antioxidant-related pathways, and that the observed increase in E_3_ was more likely attributable to the presence of the probiotic. In contrast, niacinamide levels significantly decreased from E_1_ to E_3_, with E_2_ displaying intermediate, although not statistically significant, values. As a precursor of NAD^+^ and NADP^+^, this reduction could indicate a progressive increase in NAD^+^ turnover, possibly reflecting greater metabolic demand. The intermediate profile of E_2_ suggested a partial upregulation of energy and redox metabolism induced by *Ulva*, which appeared to be further enhanced in the presence of *Phaeobacter* in E_3_.

Glycerol acts as a neutral osmolyte and an intermediate in lipid metabolism; when not esterified to fatty acids, it can be readily absorbed by passive diffusion due to its low molecular weight, and it is considered to be an effective energy source [41]. In our study, glycerol levels showed a significant decrease only in E_3_ compared with E_1_, while DMA, a product of nitrogenous osmolyte degradation, exhibited a notable increase under the same condition. This inverse relationship could reflect a shift in osmoregulatory and metabolic strategies triggered by the combined macroalgae–probiotic treatment. Specifically, the decline in glycerol could reflect reduced osmotic stress or increased utilization for energy production, suggesting a more stable physiological state in E_3_. At the same time, the rise in DMA pointed to enhanced methylamine turnover, potentially associated with the increased catabolism of nitrogen-containing osmoprotectants such as betaine. Together, these patterns suggested that fish reared under E_3_ conditions relied less on passive osmolyte accumulation and instead engaged more dynamic regulatory mechanisms, possibly mediated by gut microbiota activity, thereby supporting greater metabolic plasticity and adaptation to the enriched rearing environment. The ongoing analysis of the fish microbiome will provide information about the potential modifications that could support this hypothesis. Previous results [42,43] with turbot larvae showed, using immunohistochemistry, that a *Phaeobacter gallaeciensis* 27-4 strain (formerly *Roseobacter gallaeciensis*) introduced via live feed (rotifers) could be found in the digestive tract but did not colonize the turbot larval digestive tract. However, *Phaeobacter* strains, due to antagonistic activity, may modify the internal or external microbiome of fish.

Taurine is a sulfur-containing compound with multiple physiological roles in fish, including osmoregulation, antioxidant defense, and cellular protection [44,45]. It is considered to be a conditionally essential nutrient, particularly in marine species, which often have limited capacity to endogenously synthesize taurine and must rely on dietary intake [46]. In the context of aquaculture, taurine contributes to maintaining an ionic balance under fluctuating salinity conditions, stabilizing cell membranes, and scavenging reactive oxygen species, thereby protecting tissues from oxidative damage. It also plays a critical role in bile acid conjugation, lipid digestion, and immune modulation [47]. In our study, taurine levels were found to increase in E_3_, suggesting a physiological response to the more complex rearing environment that included both *U. ohnoi* and *Phaeobacter* sp. This elevation could reflect an enhanced antioxidant response or improved osmoregulatory efficiency, consistent with previous findings in fish subjected to sustained water flow or moderate physical stress. For instance, Wang et al. [33] reported upregulated taurine metabolism in *Micropterus salmoides* exposed to flow-induced exercise in a RAS, highlighting its role in supporting energy metabolism and reducing oxidative stress under an increased physiological demand. Whether reflecting an adaptive antioxidant mechanism or increasing metabolic turnover, taurine appears to be a sensitive biomarker of a fish’s capacity to cope with intensified environmental or nutritional inputs. Its modulation in E_3_ thus pointed to the beneficial effects of the macroalgae–probiotic combination in promoting cellular protection and metabolic resilience.

## 4. Materials and Methods

### 4.1. Experimental Design

Three growth trials followed one another, with adult Senegalese sole of approximately 85 g in individual weight received from Aquacria Arousa, S.L. (Pontevedra, Spain). Upon each of three arrivals, 180 fish were acclimatized in triplicate tanks of a multimodal RAS/IMTA-RAS hosted indoors at the Instituto Galego de Formación en Acuicultura (Niño do Corvo, Illa de Arousa, Pontevedra, Spain) for 18 to 19 days. The indoor environment was mainly lit by artificial light and secondarily lit by a window oriented north–east. To prevent fish stress and exclude the different effects of the photoperiod over the three growth trials, fish were kept in a shadow, as per commercial practice, by covering the tanks with a thick mesh. At the end of the acclimatization (T_i_), the average density was set to 6.3 kg/m^2^ and the fish were fed at a 0.83% feeding rate with commercial feed (Efico sigma 874, 3.0 mm pellet; BioMar Iberia SA, Dueñas, Spain) three times per night between 19:00 and 7:00. Afterwards, fish were grown for 42 to 43 days and the system was either left as was and used as a control (RAS; first growth trial, or E_1_), or connected to three pairs of 400 L tanks hosting U. ohnoi at a density of 0.75 g/L (IMTA-RAS; second growth trial, or E_2_), or connected to three pairs of 400 L tanks hosting U. ohnoi at the same density but previously inoculated with Phaeobacter sp. 4UAC3 (IMTA-RAS–Phaeobacter; third growth trial, or E_3_). Due to the fact that darkness enhances the maintenance of *Phaeobacter* sp. 4UAC3 and the production of tropodithietic acid (TDA) [16], light in each pair of algae tanks was managed so that, during one week, one tank remained in total darkness to allow the bacterium to thrive, while the other was exposed to light conditions (800 ± 20 µmol m^−2^ s^−1^) to allow *Ulva* growth and biofiltrate nutrients. Each week, the light/dark conditions were switched. During the third trial, two inoculations with Phaeobacter were carried out. Specifically, 6 L and 6 L of bacterial culture at concentrations of 6 × 10^7^ and 1 × 10^8^ CFU/mL were added to 1200 L of the system water, respectively, three days before and three days after the end of the acclimatization period. The inoculum was added to the tanks kept in darkness at that moment and uncoupled for three and two and a half days for the first and second inoculation, respectively. During the growth trials, the physico-chemical parameters of the water were monitored to ensure they remained within the species-specific optimal ranges [48]. Portable OxyGuard Handy probes (Farum, Denmark) were used to measure the water temperature, dissolved oxygen, and pH. Ammonia, nitrites, and nitrates were monitored using commercial kits. One fish in E_1_ and two fish in E_3_ died between T_i_ and T_f_. Further details will be included in forthcoming papers, which are currently in preparation. At the end of each acclimatization (T_i_) and at the conclusion of each trial (T_f_, six weeks later), seven fish per tank were sacrificed by qualified personnel via a 2-phenoxyethanol overdose (1 mL/L for 15 min in aerated tanks) [49], then weighed and measured. After microbial sampling, which is addressed in a dedicated paper, duplicate samples of 4 g of muscle were collected from the same anatomical region on each fish, placed in tubes, and initially frozen at −20 °C for transport to the Institute of Marine Research IIM-CSIC in Vigo (Vigo, Spain) within 48 h. All six samplings were conducted under standardized conditions to avoid molecular differences between samplings that might not have been prevented by −20 °C storage, ensuring sample comparability. Upon arrival, samples were transferred to −80 °C long-term storage. The shipment to the University of Cagliari was performed using dry ice.

### 4.2. Chemicals

Analytical grade chloroform (CHCl_3_), methanol (CH_3_OH), deuterium oxide (D_2_O; 99.9%), sodium deuteroxide (NaOD; 40 wt % in D_2_O; 99.5 atom % D), deuterium chloride (DCl; 99 at. % D), and sodium 3-trimethylsilyl-propionate-2,2,3,3-d4 (TSP) were purchased from Sigma-Aldrich (Milan, Italy).

### 4.3. NMR Sample Preparation

After thawing the muscle samples, 300 ± 30 mg was weighed from each sample and transferred into a 1.5 mL vial. Into each vial, 1 mL of a methanol (MeOH) and Milli-Q water (H_2_O) solution at a 4:1 ratio was added. Subsequently, two stainless steel grinding beads (5 mm in diameter) were introduced into each vial to aid the tissue lysis. The mixture was homogenized using a TissueLyser II (Qiagen, Milan, Italy) for 2 min at 20 Hz to facilitate metabolite extraction. Following the lysis, the samples were centrifuged at 12,000 g for 10 min at 4 °C. From the centrifuged sample, 650 µL of supernatant was collected and evaporated to dryness using an Eppendorf Concentrator Plus (Milan, Italy). The dry extract was reconstituted by adding 660 µL of deuterated water (D_2_O). The pH of the solution was adjusted to 6.20 ± 0.05 by adding deuterated hydrochloric acid (DCl) or deuterated sodium hydroxide (NaOD). Subsequently, 10 µL of a 30 mM aqueous solution (100% D_2_O) of the internal standard TSP was added. The sample was then centrifuged again at 12,000 g for 10 min at 4 °C. Finally, 650 µL of the solution was transferred into a 5 mm NMR tube.

### 4.4. NMR Spectroscopy

#### 4.4.1. NMR Spectra Acquisition Parameters

^1^H-NMR experiments were performed using a Varian UNITY INOVA 500 spectrometer (Agilent Technologies, Inc., Santa Clara, CA, USA) at 300 K. ^1^H-NMR spectra were recorded using 1D NOESY pulse sequence water presaturation with a mixing time of 1 ms, a recycle time of 3.5 for water suppression, and 512 transients at 64 K points over a spectral width of 6000 Hz (12 ppm).

#### 4.4.2. NMR Spectra Processing

^1^H-NMR spectra were processed using MestReNova software (version 14.2.1-2021, Mestrelab Research SL, Santiago de Compostela, Spain). Apodization was applied by multiplying the FID by an exponential function of 0.3 Hz. Baseline and phase corrections were manually adjusted, and the chemical shift scale was calibrated using the TSP signal and δ = 0 ppm as an internal reference. Spectral regions affected by chemical shift variations due to pH fluctuations were accordingly aligned by using an in-house modified version of Correlation Optimized Shifting (*i*Coshift) able to perform the Coshift in localized regions of the spectrum [50]. This step was performed using MATLAB^®^ (R2024b, The Mathworks Inc., Natick, MA, USA). After removing spectral regions corresponding with background noise, residual water signals, and the internal standard, spectra were normalized using Probabilistic Quotient Normalization (PQN) [51,52], a widely applied method in metabolomics to account for concentration variations (e.g., dilution effects) while preserving relative differences. After normalization, spectra were subsequently divided into bins of 0.002 ppm in width.

#### 4.4.3. NMR Metabolic Profile

Metabolite signal assignment was performed using Chenomx NMR Suite (evaluated version 8.3) and literature data [24,53,54]. A total of 36 metabolites were identified in the spectra. Among these, 30 metabolites with well-resolved and isolated signals were selected for the statistical analysis as they allowed reliable peak integration. The relative concentrations were calculated by integrating the area under each selected signal.

### 4.5. Multivariate and Univariate Statistical Data Analysis

Fish biometric data were analyzed with R 4.0.1 by assessing normality and homoscedasticity using Shapiro–Wilk and Levene’s tests followed by a one-way ANOVA.

The multivariate statistical analysis (MVSDA) of the NMR dataset was performed on the PQN and binned spectra. The mean-centered Principal Component Analysis (PCA) was selected as the reference method for comparison as it is an unsupervised approach that captures the total sample variance by projecting it into a lower-dimensional space [55]. It aims to identify potential outliers and/or the presence of clusters by analyzing both the score plot and the loading plot. All models were generated using custom algorithms implemented in R 4.0.1. For the univariate analysis (UVSDA), Jamovi software (version 2.3.28) was used [56]. The Shapiro–Wilk test was performed to assess if sets of data points were normally distributed [57], while group differences were evaluated using the Kruskal–Wallis test followed by pairwise comparisons with the Dwass–Steel–Critchlow–Fligner test. To account for multiple testing across metabolites, *p*-values from the Kruskal–Wallis tests were adjusted using the Benjamini–Hochberg False Discovery Rate (FDR) procedure. In addition to the *p*-value, the Kruskal–Wallis test provides two other important parameters, the chi-squared statistic (χ^2^) and the epsilon-squared (ε^2^). The χ^2^ value indicates the extent of differences in metabolite levels among experimental groups, while ε^2^ quantifies the effect size, representing the proportion of variance explained by group differences (ε^2^ values of around 0.01 are considered to be small, values around 0.06 indicate a moderate effect, and values greater than 0.14 are interpreted as large effects) [58,59].

## 5. Conclusions

The present study shows that the metabolomic analysis of Senegalese sole muscle can reveal metabolic changes in fish reared under three different aquaculture systems. Among these, the system incorporating the macroalga *U. ohnoi* and the probiotic bacterium *Phaeobacter* sp. 4UAC3 appeared to have the most significant impact on the fish’s energy and protein metabolism. Taken together, these findings suggest that a combination of *U. ohnoi* and *Phaeobacter* sp. (E_3_) could promote a broader and more coordinated metabolic adjustment than the macroalga alone. Notably, the intermediate profiles observed in E_2_ suggest that *Ulva* alone could influence fish physiology, particularly in relation to tissue remodeling and redox balance. However, the inclusion of the probiotic appeared to amplify these effects, leading to enhanced metabolic plasticity and potentially improved physiological performance. These results were consistent with the idea that a dynamic and moderately stimulating environment, such as that provided under the E_3_ condition, could trigger beneficial metabolic adaptations, partly similar to those reported under exercise-induced stress in fish. These effects likely involve both energy metabolism and structural remodeling, ultimately supporting greater physiological resilience and muscle function.

In summary, the synergy between *Ulva* and the probiotic actions of *Phaeobacter* in a balanced environment such as an IMTA-RAS creates optimal conditions for amino acid accumulation in aquaculture fish, hinting at possible fish welfare and performance improvements while contributing to more sustainable and efficient aquaculture practices. As this investigation represents only one component of a broader study exploring the effects of these three rearing systems on fish welfare, product quality, *Ulva* performance, the system microbiome, and water parameters, a more comprehensive understanding requires the integration of metabolomic data with microbiological, biochemical, and quality analyses. This multidisciplinary approach is essential to fully elucidate the underlying metabolic dynamics and their implications for sustainable aquaculture. It will support the development of optimized farming practices and innovative strategies to improve the efficiency and sustainability of the sector.

## Figures and Tables

**Table 1:** Biometric results of fish undergoing the three experimental conditions (E_1_: RAS; E_2_: IMTA-RAS with *U. ohnoi*; E_3_: IMTA-RAS with *U. ohnoi* inoculated with the probiotic *Phaeobacter* sp. 4UAC3).

Parameter	E_1_	E_2_	E_3_	*p*-Value
Weight gain (g)	26.0 ± 0.7	45.5 ± 15.3	43.4 ± 7.8	ns
Specific growth rate	0.61 ± 0.04	0.87 ± 0.24	0.99 ± 0.22	ns
Fulton’s condition index K	1.63 ± 0.18	1.67 ± 0.23	1.68 ± 0.14	ns

ns: not significant.

**Table 2:** Results of the Kruskal–Wallis test comparing metabolite levels identified in the ^1^H-NMR spectra of aqueous muscle extracts from *S. senegalensis* sampled at the final time point of the three experimental trials. Statistically significant *p*-values (*p* < 0.05) are shown in bold.

Metabolite	*p*-Value	ε^2^	q
Alanine	**<0.001**	0.341	**0.002**
β-Alanine	**<0.001**	0.243	**0.002**
AMP/IMP	0.239	0.046	0.265
Asparagine	0.415	0.028	0.444
Aspartate	0.063	0.202	0.076
Betaine	**<0.001**	0.270	**0.002**
Cystathionine	**<0.001**	0.370	**0.002**
Creatine	0.798	0.007	0.79
DMA	**<0.001**	0.813	**0.002**
DMG	**0.002**	0.202	0.003
Formate	**<0.001**	0.314	**0.002**
Fumarate	**0.049**	0.100	0.061
Glycerol	**0.002**	0.203	**0.003**
Glycine	**0.003**	0.203	**0.004**
Glutamine	**<0.001**	0.305	**0.002**
Inosine	**<0.001**	0.337	**0.002**
Isoleucine	**0.027**	0.118	**0.035**
Lactate	0.595	0.017	0.616
Leucine	**0.009**	0.155	**0.013**
Lysine	**0.023**	0.121	**0.031**
Malate	**0.003**	0.189	**0.004**
Methylguanidine	**<0.001**	0.478	**0.002**
Niacinamide	**<0.001**	0.284	**0.002**
Phenylalanine	**<0.001**	0.448	**0.002**
Succinate	**<0.001**	0.269	**0.002**
Taurine	**<0.001**	0.588	**0.002**
Tyrosine	**<0.001**	0.601	**0.002**
TMAO	0.232	0.047	0.265
Trans-4-hydroxy-L-proline	**<0.001**	0.390	**0.002**
Valine	**0.002**	0.121	**0.003**

q: FDR-corrected *p*-Value.

## Data Availability

The datasets used and/or analyzed during the current study are available from the corresponding author upon request.

## Supplementary Materials

The following supporting information can be downloaded at: https://www.mdpi.com/article/10.3390/molecules30122518/s1, Figure S1: Hotelling T^2^ and DModX criteria, Figure S2: Raw 1H-NMR spectra overlays.

## Abbreviations

The following abbreviations are used in this manuscript:

AMP	Adenosine monophosphate
DMA	Dimethylamine
DMG	Dimethylguanidine
FDR	False Discovery Rate
FID	Free Induction Decays
1H NMR	High-Resolution Nuclear Magnetic Resonance
IMP	Inosine monophosphate
IMTA-RAS	Integrated Multitrophic Recirculating Aquaculture System
MVSDA	Multivariate Statistical Data Analysis
PCA	Principal Component Analysis
PQN	Probabilistic Quotient Normalization
TCA	Tricarboxylic Acid Krebs Cycle
TDA	Tropodithietic Acid
TMAO	Trimethylamine N-Oxide
TSP	3-(Trimethylsilyl)-(2,2,3,3-^2^H_4_)-1-Propionate Sodium Salt
UVSDA	Univariate Statistical Data Analysis
