# Supplementary material for: Metabolic Profile of Senegalese Sole (Solea senegalensis) Muscle: Effect of Fish–Macroalgae IMTA-RAS Aquaculture
Source: Molecules. 2025 Jun 9;30(12):2518. doi: 10.3390/molecules30122518 (PMC12196385; doi:10.3390/molecules30122518)
Supplement: Supplementary file 1 [file molecules-30-02518-s001.zip › molecules-3647526-supplementary.pdf]

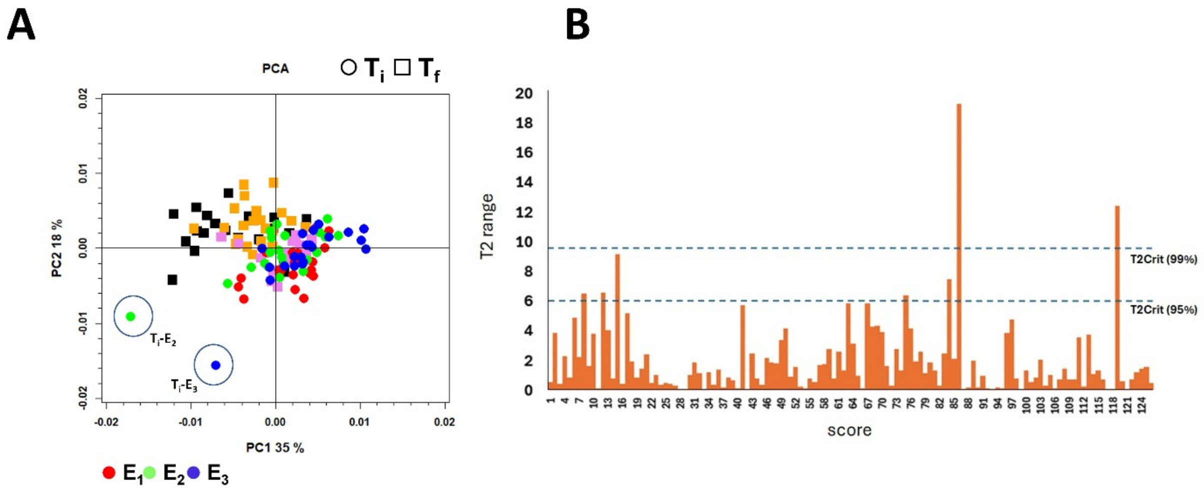

Figure S1. The two Ti spectra, circled in the score plot (**A**), were identified as outliers, and objective criteria were applied, including Hotelling T^2 and DModX, to support their exclusion (**B**).

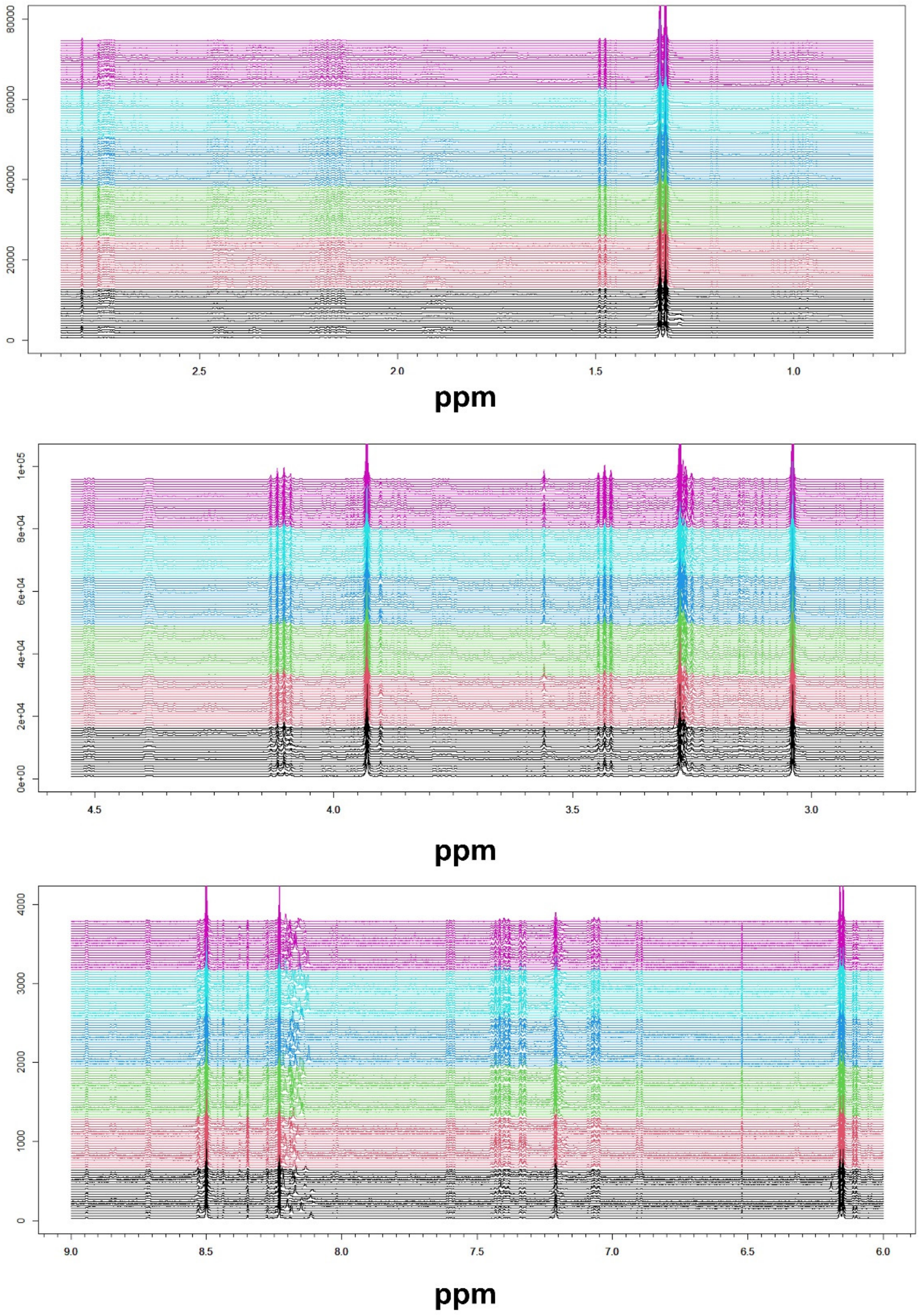

Figure S2. Raw ^{1}H-NMR spectra overlays to allow for independent assessment of signal assignments listed in Figure 1.
